# The binding interactions that maintain excitation–contraction coupling junctions in skeletal muscle

**DOI:** 10.1085/jgp.201812268

**Published:** 2019-02-06

**Authors:** Eduardo Ríos, Dirk Gillespie, Clara Franzini-Armstrong

**Affiliations:** 1 Section of Cellular Signaling, Department of Physiology and Biophysics, Rush University, Chicago, IL; 2 Department of Cell and Developmental Biology, University of Pennsylvania, Philadelphia, PA

## Abstract

Calcium for contraction of skeletal muscles is released via tetrameric ryanodine receptor (RYR1) channels of the sarcoplasmic reticulum (SR), which are assembled in ordered arrays called couplons at junctions where the SR abuts T tubules or plasmalemma. Voltage-gated Ca^2+^ (Ca_V_1.1) channels, found in tubules or plasmalemma, form symmetric complexes called Ca_V_ tetrads that associate with and activate underlying RYR tetramers during membrane depolarization by conveying a conformational change. Intriguingly, Ca_V_ tetrads regularly skip every other RYR tetramer within the array; therefore, the RYRs underlying tetrads (named V), but not the voltage sensor–lacking (C) RYRs, should be activated by depolarization. Here we hypothesize that the checkerboard association is maintained solely by reversible binary interactions between Ca_V_s and RYRs and test this hypothesis using a quantitative model of the energies that govern Ca_V_1.1–RYR1 binding, which are assumed to depend on number and location of bound Ca_V_s. A Monte Carlo simulation generates large statistical samples and distributions of state variables that can be compared with quantitative features in freeze-fracture images of couplons from various sources. This analysis reveals two necessary model features: (1) the energy of a tetramer must have wells at low and high occupation by Ca_V_s, so that Ca_V_s positively cooperate in binding RYR (an allosteric effect), and (2) a large energy penalty results when two Ca_V_s bind simultaneously to adjacent RYR protomers in adjacent tetramers (a steric clash). Under the hypothesis, V and C channels will eventually reverse roles. Role reversal justifies the presence of sensor-lacking C channels, as a structural and functional reserve for control of muscle contraction.

## Abstract (precis)

Ryanodine receptors in the sarcoplasmic reticulum and voltage-gated Ca^2+^ channels on T tubules or the cell membrane coassemble into structures called couplons. Ríos et al. use quantitative simulations to test a simple hypothesis on the organization of couplons and derive functional implications of this arrangement.

## Introduction

The contraction of striated muscles is activated by calcium ions released from the sarcoplasmic reticulum (SR) in response to membrane depolarization. In skeletal muscle, calcium release occurs at specialized structures where the membrane of transverse (T) tubules, i.e., invaginations of the plasmalemma, comes close to that of the SR. There, voltage-sensing proteins of the T membrane (Ca_V_1.1 in skeletal muscles) interact with the SR calcium release channels (also called RYRs).

**Figure 1.**
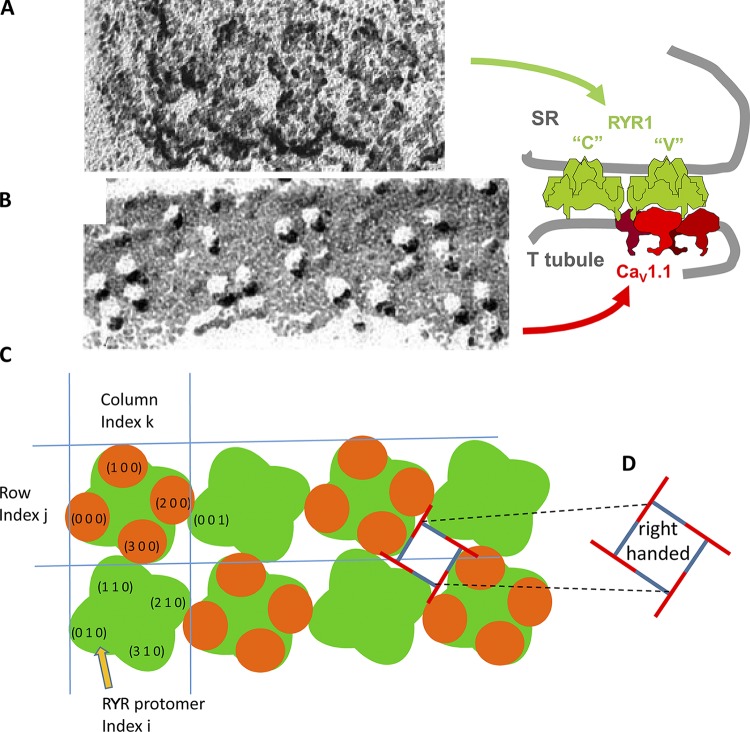
**Components of a triadic junction visible in EM images. (A)** Freeze-dried rotary shadowed junctional SR membrane from guinea pig. **(B)** Tetrads of particles (Ca_V_s) in a freeze-fractured T tubule membrane from toadfish muscle, presented with the same orientation and magnification. **(C)** Canonical couplon, with array notation (side view in side diagram). RYR tetramers (or channels, or feet; green) are identified by a row index *j* (which in T tubule couplons range between 0 and 1) and a column index *k* (0–3 in the case illustrated). Whether channels are even or odd is determined by the parity of *j* + *k*. In this canonical configuration, even channels are fully occupied by Ca_V_s (orange elements). Individual protomers are identified by index *i* (0–3), increasing clockwise within the RYR tetramer. The adjacent feet of foot (0, 0) are (0, 1) and (1, 0). In those, the adjacent protomers are (0, 0, 1), adjacent to (2, 0, 0), and (1, 1, 0), adjacent to (3, 0, 0). **(D)** Diagram to illustrate chirality or handedness in the couplon in the conventional view, which results from viewing junctions from outside the cell. We arbitrarily designate this orientation as right handed. The horizontal distance between centers of adjacent RYRs is ∼30 nm.

The spatial placement of Ca_V_s relative to RYRs, defined by Block et al. (1988), is intriguing. RYRs are homotetramers of the RYR1 protein; they comprise intramembrane domains (the channel proper) and large cytoplasmic domains with an approximately square profile in electron micrographs of the junctional gap, named “feet.” In triads of differentiated skeletal muscle, RYR tetramers cluster in orderly arrays of two rows, extending along junctional SR segments of variable lengths (from 0.2 up to 1–2 µm) that face the T tubules. In these double rows, the square profile of the foot is tilted by 22° relative to the axis of the tubule. These features are illustrated in Fig. 1 A. In cultured BC_3_H1 cells, in some types of muscle fibers, and in most fibers during differentiation, junctions are formed by association between wide SR cisternae and the surface plasmalemma (rather than T tubules), in which feet are arranged in large plaques of multiple rows (examples shown in Fig. 4). In these, the 22° tilt persists, if defined as the smallest angle between a side of the tetrad (or underlying RYR tetramer) and the line that joins centers along the row of tetrads. This angle is geometrically determined by the degree of overlap between adjacent RYR tetramers, which is the same in T tubular or peripheral junctions. The quaternary arrangement of RYR tetramers and tetrads is the same in fibers of all higher vertebrates, from bony fish up. Arrangements in various taxa are described in Di Biase and Franzini-Armstrong (2005).

Ca_V_1.1, the voltage-activated L-type calcium channels of plasmalemma and T tubules, are heteromers composed of five subunits. The main component is the α1 subunit, which contains the voltage-sensing and pore domains of the channel. Ca_V_1.1 function as the voltage sensors of excitation–contraction coupling. Ca_V_1.1 are detected in the electron microscope by the technique of freeze fracture, which reveals the position of the α1 subunit as a prominent, tall particle. In normal calcium release units (CRUs), Ca_V_1.1 particles are grouped in small clusters of four, termed junctional tetrads (Franzini-Armstrong and Nunzi, 1983; an example is in Fig. 1 B). The grouping of Ca_V_1.1 is directly dependent on isoform-specific interactions of the two skeletal muscle isoforms, Ca_V_1.1 and RYR1, as demonstrated by the fact that Ca_V_1.1 do not cluster into tetrads in CRUs of dyspedic (RYR1-null) myotubes or in cardiac myocytes where the two cardiac isoforms (Ca_V_1.2 and RYR2) are present. Tetrads consist of four Ca_V_s interacting, presumably independently of each other, with the four identical protomers of an underlying RYR1. The four components of the tetrad are precisely located slightly offset from the four corners of the interacting RYRs, so that tetrads are also skewed relative to the T tubule axis (Fig. 1).

The array of RYR tetramers, Ca_V_s, and smaller associated proteins of one junction are referred to as couplons (Stern et al., 1997; Franzini-Armstrong et al., 1999). A triad contains two couplons, one on either side of the participating T tubule. Even though Ca_V_s are also channels, the term “channels” is here applied only to RYR1 tetramers.

An unexplained detail of the interaction is that Ca_V_ tetrads abut RYRs in a skipping or checkerboard pattern, so that alternating RYR tetramers are free of sensors (Fig. 1 C). This pattern has immediate functional implications. Two classes of RYR channels, V and C, may be distinguished in a couplon (Ríos and Pizarro, 1988; Shirokova et al., 1996). V channels are the RYR channels linked to a Ca_V_ tetrad; they are presumably activated by Ca_V_s acting as voltage sensors, via conformational coupling (Nakai et al., 1996). C channels, those lacking voltage sensors, were originally envisioned as indirectly activated by calcium in a secondary response. The proposal is now disproved (reviewed in Ríos, 2018), which leaves C channels bereft of a known functional role; whether and how they participate in normal calcium release remains to be established.

The array of feet or RYR tetramers in a T tubule couplon is spatially periodic, with a period equal to the distance between adjacent feet, ∼30 nm. Over this fundamental frequency, the interacting Ca_V_1.1 superimpose the longer-range periodicity of the checkerboard (∼60 nm). The main hypothesis of the present work is that this long-range order can emerge from binary, reversible, short-range interactions between individual molecules. We propose that Ca_V_1.1–RYR1 interactions determine the checkerboard pattern, without invoking structures, interactions, or influences outside the couplon.

The hypothesis is given quantitative form in a Monte Carlo simulation. Numeric outcomes from the simulation are compared and found generally compatible with those derived from images of junctions collected by the Franzini-Armstrong laboratory. Insights from the simulation suggest extensions of the hypothesis and a role for C channels.

## Hypothesis, theory, and simulation

### Hypothesis

**Figure 2.**
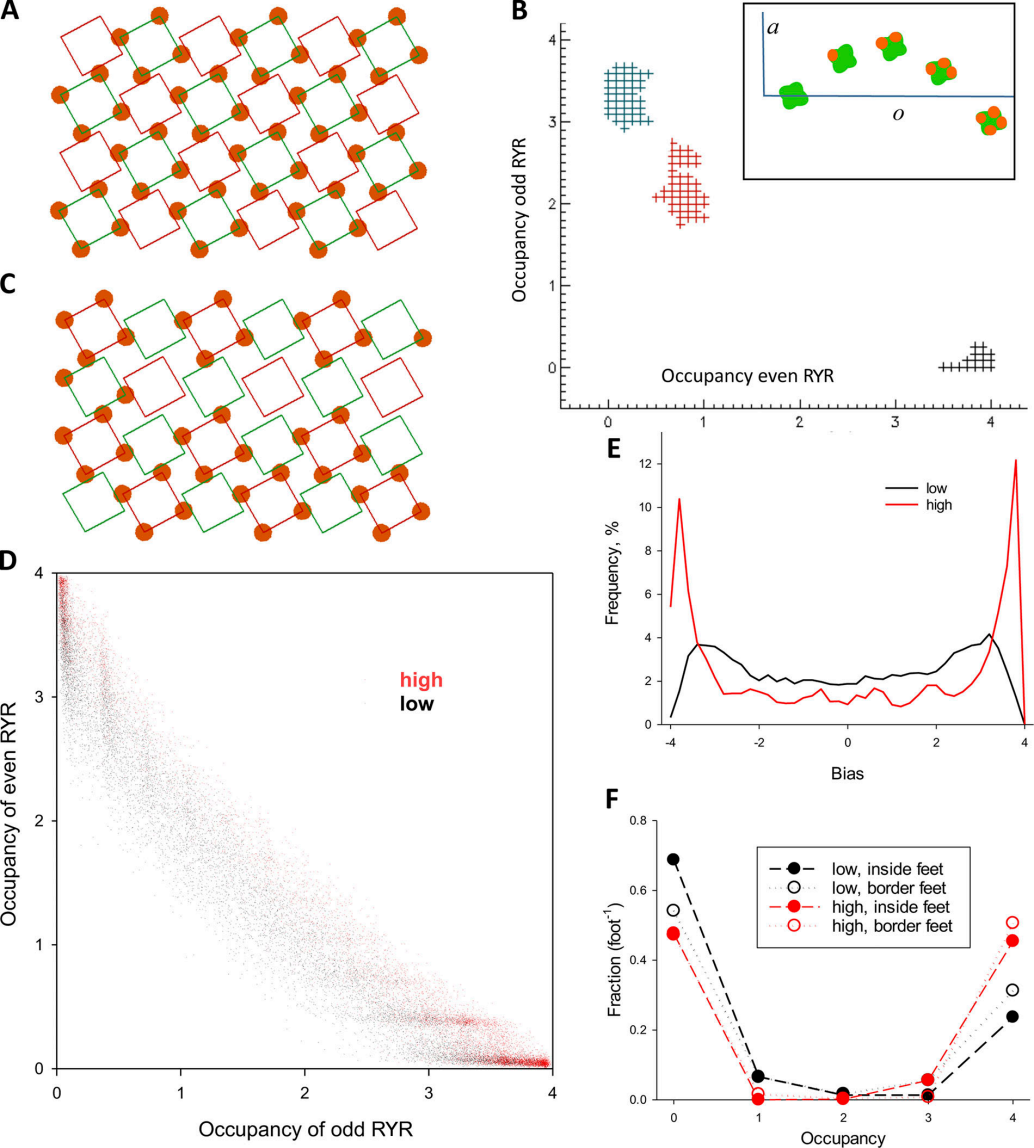
**Simulation outcomes. (A)** A couplon of four rows and six columns in canonical configuration. Green squares represent RYR1 tetramers (channels) of the V class, so named for being occupied by four voltage sensors (Ca_V_s). Red squares represent C channels, free from Ca_V_s. Green and red channels are even and odd, respectively, as determined by parity of the sum of coordinates *j* and *k*. **(B)** Evolution of the system’s configuration in simulations with *a_o_* values represented schematically in the inset. Plus symbols plot couplon occupancies of even and odd channels (*co*_e_ versus *co*_o_, defined in Eq. 8) in 2,000 successive configurations. Symbols in black represent the first set, and red and blue symbols represent configurations reached before the 10^6^ and 2 × 10^6^ configuration. **(C)** Configuration reached after 2 × 10^6^ decisions. Odd channels are largely occupied, while even channels are largely free. Full occupancy of feet (by 4 Ca_V_s) still predominates. **(D)** Couplon occupancies (*co*_e_ versus *co*_o_) averaged over 2,000 successive configurations. The plot includes 10^4^ averages, representing 2 × 10^7^ successive configurations: in black, those reached with the low parameter set (Table 1), and in red, configurations reached with the high parameter set. **(E)** Distributions of bias and difference between couplon occupancy of even and odd channels. From the distributions of bias the average asymmetry is calculated (Eq. 10). **(F)** Fractional occupancies *f*(*o*) for both sets of parameters. Open symbols plot fractional occupancies of border RYR tetramers (i.e., rows 0 and 3 and columns 0 and 5), while filled symbols represent fractional occupancies of inside tetramers.

The hypothesis requires two premises—prior assumptions warranted by observations. One is that the junctional arrangement of membranes is assured by means independent of the couplon proteins. Indeed, during muscle differentiation, SR to plasmalemma contacts are established before Ca_V_s and RYRs are present at the junction sites (Flucher and Franzini-Armstrong, 1996; Protasi et al., 1996). The establishment of junctions requires the presence of junctophilin (Zhao et al., 2011). The second premise is that the RYR tetramers self organize into regular planar arrays. Indeed, RYRs and Ca_V_s can be incorporated to the junctions independently of each other (as seen in dysgenic and dyspedic muscles). In junctions as well as in vitro, RYR channels self organize in regular two-dimensional arrays (Yin and Lai, 2000; Yin et al., 2005b), by physically interlocking an RYR tetramer with the four adjacent ones (Yin et al., 2005a).

(Note that the array has handedness, implying that the RYR tetramer is chiral. Following the conventional display, shadowed images of RYR arrays on SR membrane [Fig. 1, A and C], freeze-fracture images of Ca_V_s arrays, and V and C grids [Figs. 4, 5, and 6] are shown as viewed from outside the cell. In that case, we call the orientation right handed, represented diagrammatically by Fig. 1 D. By contrast, the computer-generated grids in Fig. 2 and in Figs. S1, S2, S3, and S4 have the left-handed orientation of couplons viewed from within the SR.)

The hypothesis has three components: (1) All RYRs of isoform 1 are identical; C and V channels are defined exclusively by their relationship with Ca_V_s. (2) The skeletal muscle couplon is a mechanically linked continuum; all interactions involve physical contact. (3) The checkerboard structure of the skeletal muscle couplon depends only on forces between couplon molecules, free of other constraints.

The hypothesis is formulated in terms of particles, rather than molecules. This is done for two reasons: There is no firm information on specific roles by individual molecules or regions within molecules that define binding stability. Additionally, the data against which the hypothesis can be tested are the distributions of particles visible in electron microscopic images. Albeit the compositions of these particles are multimolecular, they are referred to as RYRs (protomers or tetramers) and Ca_V_s (individually or in Ca_V_ tetrads).

### Theory

The hypothesis is quantitatively formulated in terms of interaction energies. The formulation, together with example values for the parameters (energies), is described here in reference to Fig. 1 C, which represents a couplon of two rows containing four RYR tetramers in each row. The four RYR protomers in every tetramer are identified by index *i* (0–3), rows are identified by index *j*, and columns (in this case, pairs of RYR tetramers) are identified by *k*; thus, every RYR tetramer in the couplon is identified by index vector (*j*, *k*), and every protomer is identified by vector (*i*, *j*, *k*).

In the model, adjacency is a critical property. In the couplon of Fig. 1 C, every RYR tetramer is adjacent to two or three others. Thus, tetramer (1, 0) is adjacent to (0, 0), (1, 1), and (0, 2). In couplons with more than two rows, included in simulations below, an RYR tetramer is adjacent to up to four others. Adjacency also refers to pairs of RYR protomers in different tetramers; thus, protomers (3, 0, 0) and (1, 1, 0) are adjacent.

Every RYR protomer is a candidate site for binding of Ca_V_1.1. Binding is regarded as a two-body event, determined by a single energy difference that subsumes all interactions among molecular components of the two particles.^1^

Every protomer has two possible states, occupied (bound to a Ca_V_) or free. The energy of the whole system (the couplon) is the sum of energies of its individual RYR tetramers; in turn, the energy of individual RYR tetramers depends solely on the occupancy of its four protomers and their adjacent protomers.

Full description of the model therefore requires specifying the energy of a tetramer with zero to four bound Ca_V_s. Because the hypothesis admits that these energies will depend also on simultaneous occupancy of an adjacent protomer, the energies change according to the state of the adjacent protomers.

The sets of RYR tetramer energies are conveniently represented with vectors (indexed arrays of numbers), A–E:

$$\begin{array}{l} A=\left[a_{o}\right],\;o=0-4\\ B=\left[b_{o}\right],\;b_{o}=a_{o}+S,\;o=1–4\\ C=\left[c_{o}\right],\;c_{o}=b_{o}+S,\;o=2–4\\ D=\left[d_{o}\right],\;d_{o}=c_{o}+S,\;o=3–4\\ E=\left[e_{o}\right],\;e_{o}=d_{o}+S,o=4. \end{array}$$ (1)

The indexed symbols in brackets represent the multiple components of the arrays. The *a*_o_ are the energies of a foot occupied by *o* (= 0 – 4) Ca_V_s. The *b*_o_ are the corresponding energies of the foot when one of its protomers and its adjacent protomer are simultaneously occupied; they differ from the *a*_o_ by the additive constant *S*. The *c*_o_, *d*_o_, and *e*_o_ are the energies when two, three, or all of its protomers and adjacent protomers, respectively, are occupied.

Because the configurations defined by these rules only depend on energy differences, the initial energy *a*_0_ is arbitrary and is set to 0. The model thus has five adjustable parameters, *a*_1_–*a*_4_ and *S.* Because the energies in B assume occupancy of at least one protomer, B is a four-element vector (*o* in *b*_o_ cannot be 0). Similarly, the dimensions of vectors C–E are successively reduced to 3, 2, and 1. Specifically, E has a single member, *e*_4_ = *a*_4_ + 4*S*.

In the canonical checkerboard structure (Fig. 1 C), there are no particles on adjacent protomers belonging to separate tetramers (an assumption that reflects the absence of particles in close contact in the images). This is enforced in the model by a positive value of *S*, relatively large compared with other energies. This rule implements the mechanism proposed by Protasi et al. (1997) to justify the alternate occupancy of feet in the canonical couplon. It was formulated originally as steric hindrance; simply, a bound Ca_V_ extends beyond the limits of the underlying foot, making it difficult to place another Ca_V_ on the adjacent protomer of the adjacent foot. *S* equals the energy penalty assigned to reflect the steric effect. An example in Fig. S1 illustrates the gross inadequacy of a model without this effect (i.e., *S* = 0).

The energies *a_o_*, *o* = 1 – 4, define how occupancy of one protomer affects the binding affinity of other protomers in the same tetramer. Choosing values *a_o_* that make binding at one site independent of the state of the others also had outcomes that differ widely from the observations (illustrated in Figs. S2 and S3 and Table S1). We found that the checkerboard arrangement of tetrads could only be approximated by models that combined a strong steric effect (established by a high value of *S*) with an allosteric effect, or positive cooperativity, whereby binding at one protomer was favored by binding to the other protomers in the same tetramer.

A first example with both properties assumes the parameter values in the low entry of Table 1: A = [0, 3, 4, 2, −2] and *S* = 6. To make energies dimensionless in these implementations, energies are simply divided by *kT* as they would be in a Boltzmann factor. The energies of this example are graphically represented in the inset of Fig. 2 B.

**Table 1.** Model parameters

**Parameters**	**Occupancy**					**Steric term**
	**0**	**1**	**2**	**3**	**4**	
**Energy per tetramer**						
Low	0	3	4	2	−2	6
High	0	3	4	1	−3	8
Low, less affinity	0	4	5	3	−1	6

“Low,” “high,” and “low, less affinity” identify the three sets of parameters used in the simulations discussed in the text. Other sets are used in simulations documented in Online supplemental material.

With parameters specified, the energy of every configuration can be calculated. Examples follow: the energy of RYR tetramer (0, 0) will depend on the number and location of its occupied protomers as well as the occupancy of the adjacent protomers, namely, (1, 1, 0) in adjacent tetramer (1, 0) and (0, 0, 1) in tetramer (0, 1). If the adjacent protomers are free, the energy of tetramer (0, 0) will be the corresponding *a*_o_, that is, 0, 3, 4, 2, or −2, corresponding to *o* = 0–4. The energy decreases as occupancy increases to 3 and 4; this decrease implements the allosteric interaction (positive cooperativity).

If protomer (1, 1, 0) is occupied, the steric effect may change the couplon energies. If zero to three of the protomers in (0, 0) are occupied but protomer (3, 0, 0) remains free, the energies will still be the *a*_o_, that is, 0, 3, 4, 2 (*o* = 0–3). When protomer (3, 0, 0) is occupied, the energies will be the *b*_o_, namely, 9, 10, 8, and 4 (*o* = 1–4; Table 1). The sets of values [*c*_o_], [*d*_o_], and [*e*_o_] (consisting of the single value *e*_4_) apply at higher levels of occupancy of adjacent protomers in adjacent RYR tetramers.

### Simulation

The observable features of the structure defined by the above theory were determined by Monte Carlo simulation. The simulation assumes the existence of an array of RYR tetramers that remains stable throughout, while individual protomers may bind Ca_V_s in a reversible reaction.

The simulation consists of sampling the space of possible couplon configurations. This is implemented by *N* successive stochastic decisions at individual RYR protomers, each leading to a new configuration. For a free protomer the decision (to bind or remain free) depends on the probability factor

$$\begin{array}{l} p_{B}=e^{-\left(E_{Bound}-E_{Free}\right)}\\ =e^{-\left(\Delta E\right)}. \end{array}$$ (2)

The exponent is the difference Δ*E* between the energies of the entire couplon with the specified site bound or free. Given the simple way in which energies are defined, Δ*E* depends only on the occupancy of the other protomers in the same RYR tetramer as well as the simultaneous occupancy of the adjacent protomers in the adjacent RYR tetramers.

**Figure 3.**
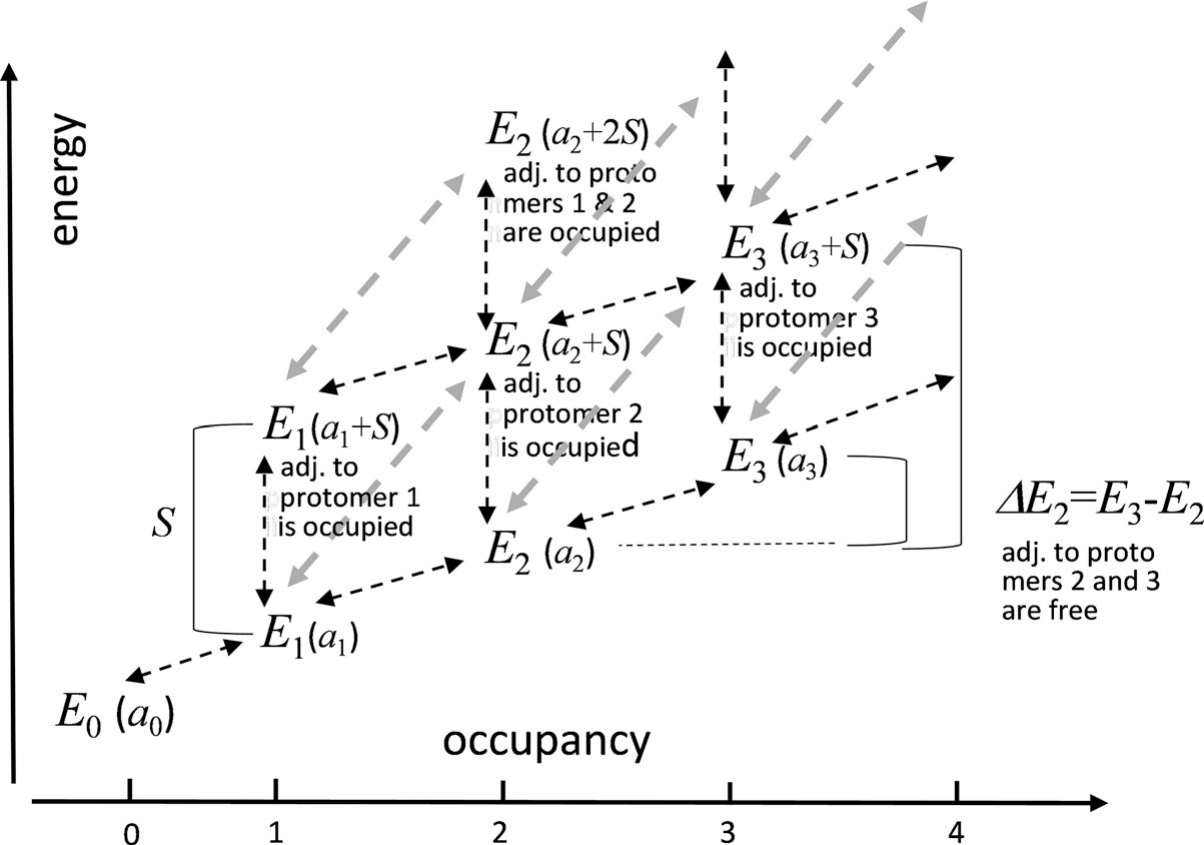
**State diagram of one RYR tetramer to illustrate calculation of binding energies Δ*E*.** The states are identified by their energy *E_o_* (for *o* = 0–3), expressed in parentheses in terms of the model parameters. As shown, the tetramer energy depends on its occupancy *o* and on simultaneous occupancy of adjacent protomers in adjacent tetramers. *E_o_* may therefore adopt multiple values for the same occupancy. Among the many states available to a tetramer, the diagram illustrates those with four of five possible *o* values and up to two simultaneously occupied adjacent protomers. Arrows represent transitions.^2^ The diagonal gray arrows represent binding to (or unbinding from) a protomer that has the adjacent protomer occupied. In this example, the lower value of *ΔE*_2_ illustrates a case of Eq. 3 and the higher value illustrates Eq. 4 with starting occupancy *o* = 2 in both cases. Parameters *a*_o_ and *S* have arbitrary values, which result in positive *ΔE*_2_; other possibilities are explored later.

The calculation, illustrated for some values of the occupancy in Fig. 3, requires knowledge of the current state. For a binding decision at an initially free RYR protomer, the following considerations apply. If the adjacent protomers are free, the difference Δ*E* may adopt one of four values, depending on the initial occupancy *o*,

$$\Delta E_{o}=a_{o+1}-a_{o},\;o=0–3.$$ (3)

In other words, the energy difference results from the increase by 1 of the occupancy of an RYR tetramer free of steric effects (transitions represented by black oblique arrows in Fig. 3). If instead the adjacent protomer is occupied, the calculation will involve energies *a* and *b* (gray arrows):

$$\Delta E_{o}=b_{o+1}-a_{o},o=0-3.$$ (4)

This rule applies whether or not more adjacent protomers are occupied producing additional steric clashes. Refer to Fig. 3 for more details.

Having determined Δ*E*, the decision is reached according to the Metropolis–Hastings algorithm (Chib and Greenberg, 1995). Namely, if Δ*E* is negative, the change in state takes place. If Δ*E* is positive, the transition is treated as a random event with probability $e^{-\Delta E}$. The process is implemented numerically by drawing a random number *r* uniformly distributed in the range [0, 1] and allowing the transition if and only if $r<e^{-\Delta E}$.

When the RYR protomer where the decision takes place starts occupied and the adjacent protomer is free, the calculation of the difference in energy is modified as follows:

$$\Delta E_{o}=a_{o-1}-a_{o},\;o=4-1.$$ (6)

If the adjacent protomer is occupied,

$$\Delta E_{o}=a_{o-1}-b_{o},\;o=4-1.$$ (7)

The possible transition in this case is dissociation of the bound Ca_V_. The rules for deciding whether it takes place remain the same, requiring a random decision only if Δ*E* is positive.

While our main goal is to justify the structure of the two-row couplons in T tubule junctions, large sets of images that can be compared with the simulations have been obtained from BC_3_H1, a line of skeletal muscle origin (Marks et al., 1989, 1991) and from frog *cruralis* muscle. Both have extensive planar junctions at the surface membrane, containing couplons with multiple rows of RYR channels (Franzini-Armstrong, 1984; Protasi et al., 1997).

A simulation is illustrated in Fig. 2. The couplon, with four rows and six columns, is shown in its canonical configuration in Fig. 2 A (the orientation here is left handed, which has no consequences for the simulation). Green and red squares represent V and C channels, respectively. Energies *a*_o_ are represented in the inset of Fig. 2 B. The simulation consists of sampling the configuration space via successive decisions on randomly selected RYR protomers. Additional configurations are accumulated until convergence, that is, when the distributions of emergent measures become stable. Because the locations of bound Ca_V_s change, a nomenclature that describes the location of the RYR tetramers (or channels) within the couplon is necessary. We call the RYR tetramers in green even and the others odd, as determined by the parity of the sum of their indexes.

Five measures emerge from the simulations as state variables comparable with experimental observations.

Occupancy, *o*(*j*, *k*), a property of individual RYR tetramers, is the number of RYR protomers occupied by Ca_V_s; it adopts integer values between 0 and 4. In the canonical configuration *o* is 4 for even and 0 for odd tetramers.

Couplon occupancy (*co*), a property of individual configurations, is occupancy averaged over all RYR channels in the couplon; unlike *o*, which takes only integer values, it may adopt any value between 0 and 4. Let *n_t_*, *n_e_*, and *n_o_* represent respectively total, even, and odd numbers of channels; *n_t_* = *n_e_* + *n_o_* . Let *n_r_* and *n_c_* represent number of rows and columns of the couplon, then

$$co=\sum_{j=1}^{n_{r}}\sum_{k=1}^{n_{c}}o\left(j,k\right)/n_{t}.$$ (8)

It is useful to restrict the averages to even or odd channels,

$$co_{e}=\sum_{j}\sum_{k,\left(k+j\right)\in 2Z}o\left(j,k\right)/n_{e}$$

$$co_{o}=\sum_{j}\sum_{k,\left(k+j\right)\in 2Z+1}o\left(j,k\right)/n_{o},$$ (9)

where 2*Z* represents the even integers set. With these definitions, *co* is the average of odd and even couplon occupancies. In the canonical configuration, *co* = 2, *co*_e_ = 4, and *co*_o_ = 0.

Couplon occupancy is averaged over a number *N* of successive configurations. As *N* increases, the distributions of configurations and emergent variables become stable. At this point, average couplon occupancy and other state variables become suitable for comparison with the average observations on images.

Fractional occupancy, *f*(*o*), a property of individual configurations, is the fraction of channels in the couplon with occupancy *o*. Again, *f* can be restricted to even or odd channels (*f*_e_, *f*_o_), and their averages over a large number of configurations can be compared with observations. In the canonical configuration, *f*(4) = 0.5 and *f*(0) = 0.5 and 0 for other values of *o*; *f*_e_(*o*) = 1 for *o* = 4 and 0 otherwise, while *f*_o_(0) = 1.

Bias (*g*), also a property of individual configurations, is the difference between couplon occupancies of even and odd channels. It adopts any value between −4 and 4. Bias is not comparable with observations because a distinction between even and odd channels is not feasible in images.

Asymmetry (*y*), the absolute value of bias, is instead comparable with observations.

$$y=\left|co_{e}-co_{i}\right|.$$ (10)

In the canonical configuration, *g* and *y* equal 4.

In simulations with couplons of more than two rows we also recorded separately the occupancies at border channels (*j* = 0 or 3, *k* = 0 or 5 in the example) and inside channels. It was possible then to compute the state variables defined by Eqs. 8 and 9 separately for these two sets of channels, to allow further comparisons with images of junctions. *n_tb_*, *n_eb_*, and *n_ob_* will represent the numbers of channels, total, even, and odd, in the border region; the corresponding numbers in the inside region will be denoted as *n_ti_*, *n_ei_*, and *n_oi_*.

In the case illustrated in Fig. 2, the distributions of *co*, *f*(*o*), *g*, and *y* became stable for a sample of *n* = 2 × 10^7^ configurations.^3^ Fig. 2 B illustrates the process of successive decisions and evolution of state variables (couplon occupancies *co*) for a run that started from the canonical configuration. Every symbol plots the couplon occupancy of even channels (*co*_e_), in abscissa, versus that of odd channels (*co*_o_) averaged over 2,000 successive configurations. Three groups of plus symbols represent averages in successive series of 2,000 configurations. The black symbols represent the first series; the state variables remained fairly stable, with *co*_e_ near 4 (nearly every even channel at occupancy 4) and *co*_o_ ∼0. As the run continued, the occupancies changed approximately reciprocally (increasing at odd and decreasing at even channels). The symbols in red and blue in Fig. 2 B plot *co* after 10^6^ and 2 × 10^6^ decisions, respectively.

Fig. 2 C illustrates the configuration reached after decision 2 × 10^6^. Even and odd channels exchanged features: odd channels were mostly occupied, while even channels were largely free. This outcome, trivially derived from the identical nature assumed for V and C channels, justifies the positional distinction of even and odd channels.

To illustrate the set of configurations visited, Fig. 2 D plots *co*_e_ versus *co*_o_ averaged over 2,000 consecutive configurations for all configurations reached in the run of *n* = 2 × 10^7^. A set like this one is convergent, in that the emergent distributions become stable, and the average state variables are nearly the same in every sample of the same size.

The *co* values represented by black symbols in Fig. 2 D were obtained using the "low" parameters (Table 1). Red dots plot values obtained with the parameter set listed as "high" in Table 1. This set increases the allosteric effect by upping the energy reward of occupancies 3 and 4 and enhances steric repulsion by increasing the penalty of binding at adjacent protomers.

A salient property of the distributions illustrated is the reciprocal relationship between occupancies of even and odd RYR channels; when even channels are highly occupied, odd channels are largely free, and vice versa. This property is quantified by bias, the difference in occupancy between even and odd channels (Eq. 10). The distributions of bias in the two simulations are shown in Fig. 2 E. A preference for high bias values is present in both distributions but is more marked for the high parameter set (red).

Additional emergents suitable for comparison are the fractional occupancies *f*(*o*), represented in Fig. 2 F. *f* is a five-valued function of occupancy. Occupancies of 0 and 4 are predominant, more markedly so with the high parameter set. An alternative implementation of the allosteric effect illustrated in Fig. S4, whereby the positive cooperativity of binding is present for every increment in occupancy, not just between 2 and 4, produced results incompatible with the observations.

While the switch to high resulted in a notable change in bias distribution (Fig. 2 E), *f*(*o*), which already favored occupancies 0 and 4 with the low parameters, changed much less. High values of *f*(4) in configurations of low bias reflect similar occupancies in odd and even channels, with *o* values of 4 or 0 still predominant at both locations. Extrapolated to observations, this property of the model suggests that the complete tetrads found in junctions might not necessarily be placed on alternating RYR channels. Apparent irregularities in T tubular junctions (described below) have various possible explanations; one is this feature of the simulations.

The simulations were repeated for a couplon of two rows and six columns. Quantitative outcomes are listed in Table 2 for both couplon geometries and three parameter sets. Given the simplicity of the model, these examples plus those illustrated in online supplemental material are sufficient to identify the region of parameter space that matches qualitatively the main features of the observations.

**Table 2.** Comparison of model outcomes and quantitative summaries of observations

**Couplon geometry**	**Simulations**					**Observations**			
	**4 columns × 6 rows**		**2 columns × 6 rows**			**Surface average, SEM**	T **tubules**		
Interaction energies	Low	High	Low	High	Low, less affinity		Wild type		*Stac3*−/−
Couplon occupancy	1.62	1.81	1.66	1.84	1.41	1.32, 0.06	1.24	1.70	1.26
Couplon occupancy inner feet	1.53	1.84	n.a.	n.a.	n.a.	1.71, 0.12	n.a.	n.a.	n.a.
Couplon occupancy border feet	1.66	1.76	n.a.	n.a.	n.a.	1.15, 0.08	n.a.	n.a.	n.a.
Full occupancy/feet	0.35	0.40	0.36	0.51	0.31	0.35, 0.02	0.30	0.31	0.12
Asymmetry	2.18	2.90	2.21	2.79	1.80	2.45, 0.13	2.10	n.a.	n.a.

**Figure 4.**
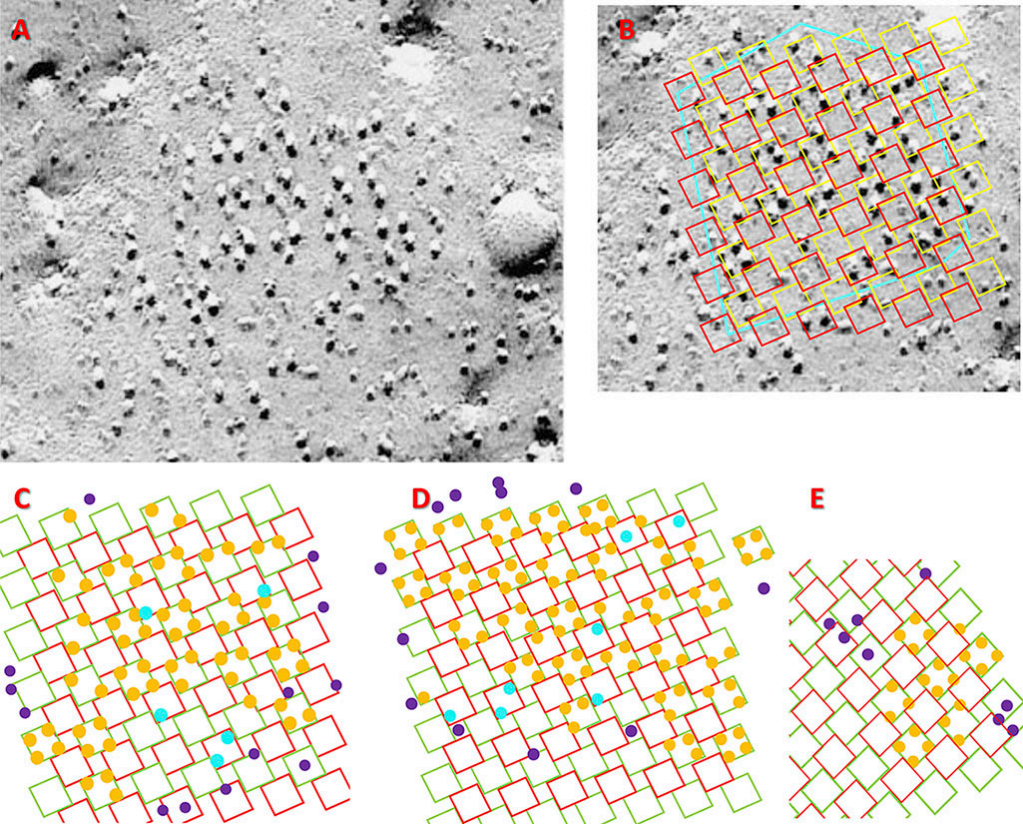
**Alignment and idealization of junction images. (A)** EM freeze-fracture image of a surface junction with vertical shading. **(B)** Illustration of the alignment stage in quantitative analysis. A grid representing the underlying array of RYR channels is superimposed to the image in A. Yellow squares represent V channels, and red squares represent C channels. The cyan polygon traces the putative borders of the couplon. **(C)** The outcome of idealization of the image in A. Orange circles represent Ca_V_s interacting with V channels, cyan circles are Ca_V_s interacting with C channels, and purple circles are particles that cannot be placed in interactions with RYR channels. **(D and E)** Additional examples of successful analysis. C and D are obtained from junctions in BC_3_H1 cells. E is from an image of a frog surface junction. The spatial scale is provided by the tetrads, which are ∼30 nm on each side.

Couplon occupancy in simulations is the variable *co* (Eq. 8) averaged over 2 × 10^7^ configurations. In observations it is calculated dividing the number of particles by the total number of tetramers in the couplon. The next two rows in the table limit the calculation to inner or border tetramers. Full occupancy/tetramer in simulations is the variable *f*(4); in observations, it is the ratio of number of complete tetrads over number of feet or tetramers contained within the couplon borders in the alignment grid. Asymmetry in simulations is the average over the same 2 × 10^7^ configurations of the variable *y* (Eq. 10). In observations it is the difference between average occupancy of V and C feet (yellow and red squares in Fig. 4 B). Values for T tubules are calculated by our method (left column under WT) or taken from the study by Linsley et al. (2017) of WT (right column under WT) and *Stac3*−/− zebrafish muscles. n.a., not applicable.

### Model versus observations

The simulations were implemented for comparison with two types of imaged couplon structures. A model with four rows produced outcomes comparable with the freeze-fracture images of multirow surface junctions of frog cruralis and BC_3_H1 cells in culture (Franzini-Armstrong, 1984; Protasi et al., 1997). A model with two rows was compared with images in T tubules of three species of fish, acquired by Franzini-Armstrong and Nunzi (1983), Block et al. (1988), Paolini et al. (2004), and Linsley et al. (2017). In all cases, the analysis was initiated by a visual identification of the presence and distribution of Ca_V_ clusters in the form of either complete tetrads or tetrads missing one or more components. This preselection rests on the assumption that a well-ordered array of RYRs underlies the imaged group of particles.

Three limitations of the freeze-fracture technique must be kept in mind when considering the import of imaged structures. The first is that Ca_V_s are never seen in direct conjunction with RYRs of the same couplon. We assume that an ordered array of Ca_V_ tetrads must be associated with an ordered array of RYRs, and thus, by selecting images where tetrads are in apparent order we can increase the probability that the images belong to complete arrays of both components, while reducing that of incomplete matches. However, even within ordered arrays tetrads are never totally complete. This can be due to several factors. When the membrane is fractured, Ca_V_s partition unequally; the majority form a particle on the cytoplasmic leaflet, but others, a minority, are absent from the cytoplasmic leaflet but appear on the luminal leaflet of the membrane. This effect is quite variable and affects the completeness of the tetrad arrays, so that by examining the cytoplasmic leaflet we are likely to underestimate the complement of appropriately placed Ca_V_s. Additionally, individual particles may be missing because the Ca_V_ was inappropriately fractured and displaced laterally by the fracturing process; this phenomenon justifies the idealization, described below, necessary to return the particle to the presumed appropriate position.

### Surface junctions

**Figure 5.**
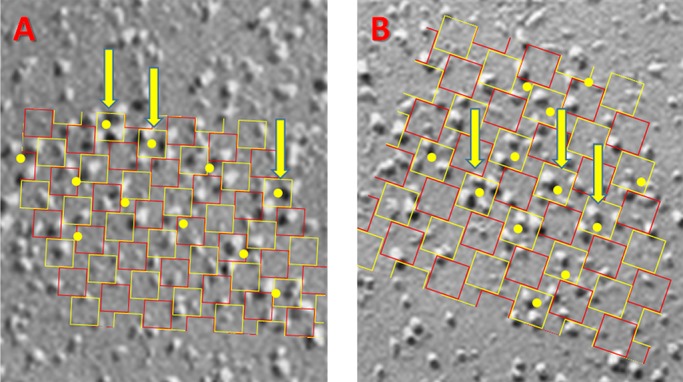
**Examples of surface junction images that could not be aligned.** A and B are representative images in different cells. Yellow dots were placed by an experienced observer to mark possible clusters of Ca_V_s attached to an underlying RYR. The grid representing the putative RYR array was scaled and rotated to align with the clusters marked by arrows, deemed to be in canonical configuration (i.e., constituting well configured tetrads, oriented parallel to one or more neighbors). In all cases, most of the other identified clusters were not aligned with the grid, due to rotation or spatial shift. Images like these were excluded from the numerical averages. Tetrads are ∼30 nm on each side.

The analysis of surface images is illustrated in Figs. 4 and 5. The image in Fig. 4 A is of a fractured membrane of a cultured BC_3_H1 cell. It has an array of well-aligned and mostly complete tetrads, oriented with its shadowing in the vertical direction. The analysis consists of two stages: alignment and idealization. Alignment, illustrated with Fig. 4 B, consists of placing a grid of squares (yellow in Fig. 4 B) that model the contours of a perfect right-handed array of RYR tetramers, so that it overlaps as many tetradic groups of particles—Ca_V_ tetrads—as possible. The size and spacing of squares in the grid are defined by a single parameter (size of squares) because RYR tetramers are tightly packed (they touch) in the assumed array. Therefore, alignment must be accomplished adjusting just two parameters, scale and angular orientation. Fig. 4 B illustrates a successful alignment: the yellow grid overlaps complete or partial tetrads of particles, presumably Ca_V_s on V channels; squares in red represent the locations of C channels, which have no bound Ca_V_s in the canonic couplon. Except for the spatial shift, yellow and red grids are identical.

The second stage in the analysis is idealization, illustrated with Fig. 4 C. It consists of placing (orange) circles representing Ca_V_s, at allowed locations, namely the corners of the V channels, consistent with the particles found in the actual image. This is an idealization for requiring in most cases some shifting of the representative circles from the original particle locations. As done in the published literature, our idealization also assumes that small bulges visible in images, which complete tetrads or orthogonal triads, are Ca_V_s broken by the membrane fracture.

Some particles appear to be located on C channels, represented by the red squares. Those particles are represented by cyan circles. The particles that cannot be clearly related to either class of channels are represented by purple circles. They appear mostly in the periphery of the well-organized areas and might be Ca_V_s or other proteins. Fig. 4 C illustrates the structure that results from idealization of the image in Fig. 4, A and B. Fig. 4 D is the idealization of another image from a BC_3_H1 cell; Fig. 4 E is the idealization of a peripheral junction in frog cruralis.

The idealizations in Fig. 4 could be done with confidence because an initial analysis indicated a high degree of order, and the alignment of Ca_V_ tetrads with putative channels (yellow squares) in the grid could be achieved together with placement of the particles at the corners of the squares representing putative RYR channels. Idealization was possible in five of eight high-resolution images from BC_3_H1 cells and two of four high-resolution images from frog cruralis, similarly preselected to indicate the presence of a complete array of RYRs. In every case, a putative couplon border was traced on the images (cyan contour in Fig. 4 B). From the idealizations we derived couplon occupancy, differences in occupancy between V and C channels, and fractional occupancies. Additionally, grouping feet in border and inside categories permitted a separate calculation of these variables in the two regions.

These outputs from observed images are comparable with simulation outputs *co*, *co_i_*, *co_b_*, *y* (asymmetry), and *f*(*o*). All are listed in Table 2. Starting with couplon occupancy, its value in surface junctions (1.31) is somewhat lower than in the simulations, but the discrepancy disappears if the analysis is restricted to inside RYR channels (1.71 observed, versus 1.53 or 1.84 with low or high parameter simulations). The observed frequency of full tetrads (0.35) and average difference in occupancy between V and C channels (2.45) are also in good agreement with the values of *f*(4) and *y* derived from the simulations.

Fig. 5 shows examples of images that could not be evaluated with this method. While some tetrads (marked with yellow dots) were visible and occasionally arranged in groups of two or three, there was no obvious extension of an orderly arrangement over the wider area. Accordingly, a grid sized and oriented to match the best defined tetrads (arrows) failed to overlap the other marked groups and match their angular orientation. This situation might result from incomplete couplon formation, distortion by the preparation process, or other causes described in the next section. The mismatch precluded any quantification that could be compared with the simulations; even in these cases, individual tetrads or triads of particles were present, with reproducible size and orthogonal arrangement.

### T tubules

**Figure 6.**
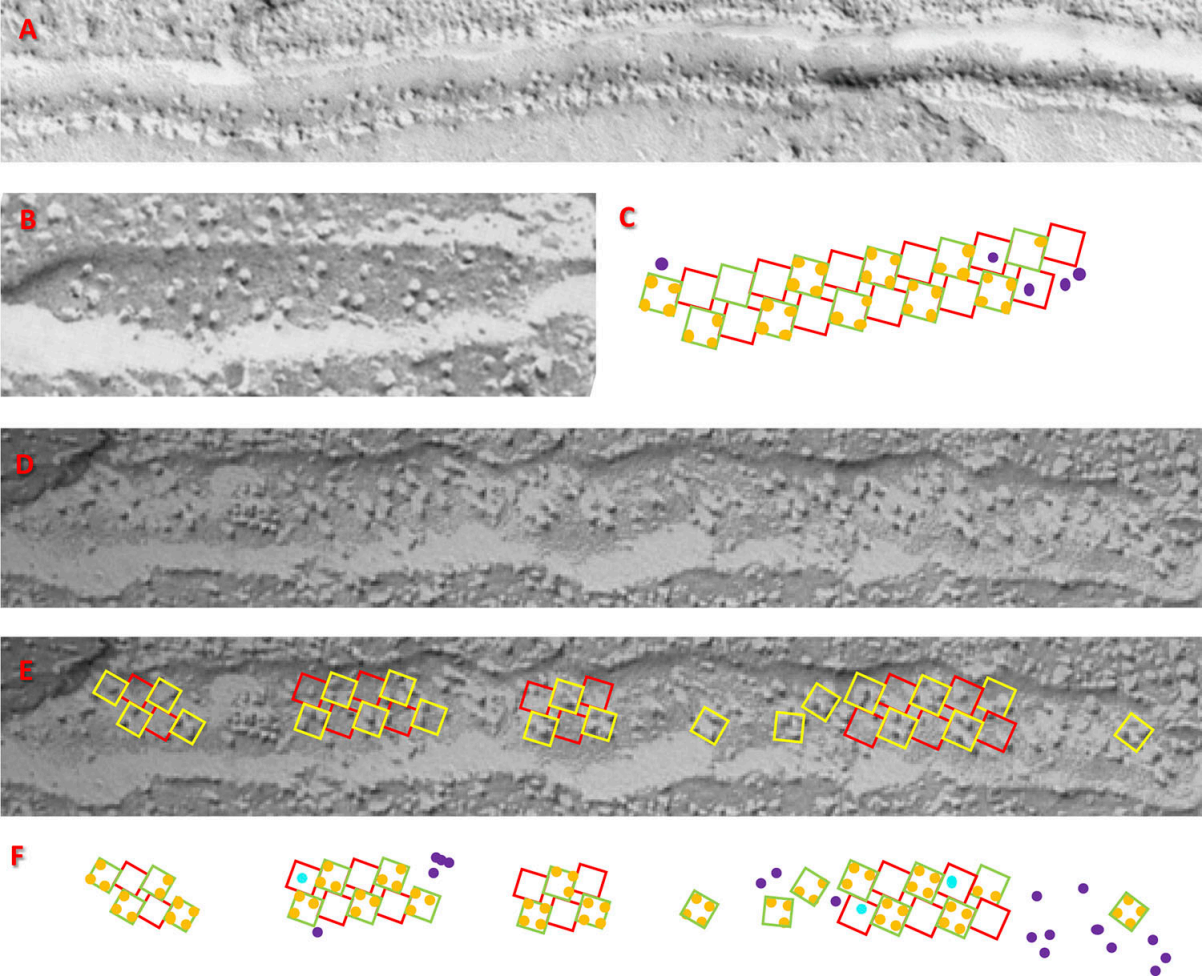
**Quantitative analysis of triadic junctions. (A)** T tubule of a toadfish swim bladder (Franzini-Armstrong and Nunzi, 1983). The quantitative analysis is not possible, as the couplon or couplons are only partially imaged. **(B)** A T tubule from toadfish, shown as one of the first examples of the canonical structure (Block et al., 1988). **(C)** Successful idealization of B. **(D)** Junction in a *stac3*^−/−^ zebrafish embryo rescued by expression of stac3 (Linsley et al., 2017). **(E)** Alignments using grids with slightly different rotation angles to segments of the tubule, consistent with the presence of small couplons separated by nonjunctional T tubule. **(F)** Idealization of the particles in D.

A similar analysis was performed for fracture images from T tubules. The analysis was limited by the fact that the T tubule membrane is curved so that extensive views of the tetrad arrays are infrequently obtained. Fig. 6 exemplifies images from multiple sources and illustrates some of the problems found in their analysis. Fig. 6 A is from the toadfish swim bladder, in the first study that revealed tetrads (Franzini-Armstrong and Nunzi, 1983); in this case, only one of the two rows of feet is visible, which makes this type of image not suitable for quantitative analysis. Fig. 6 B is from a later study of the toadfish swim bladder (Block et al., 1988); Fig. 6 C shows the result of the alignment/idealization approach to the image in Fig. 6 B. Fig. 6 D is a junction in a *stac3*^−/−^ zebrafish rescued by expression of WT stac3 (Linsley et al., 2017). In this case, the superposition could be done on separate short stretches, indicating short couplons, interrupted by segments of nonjunctional T tubule.

The measures of occupancy obtained as described above were compared with outcomes of two-row couplon simulations. Because this analysis of T tubule images was possible in a limited number of cases, we also compared with numbers derived by Linsley et al. (2017) in a study of zebrafish muscle. These numbers are listed in Table 2; the comparison shows an approximate agreement between simulations and observations.

The study of Linsley et al. (2017) offered an additional target for simulation as it provided measures *co* and *f*(4) in both WT and *stac3*^−/−^ embryos. Stac3, which binds to Ca_V_1.1 (Campiglio and Flucher, 2017; Wong King Yuen et al., 2017; Campiglio et al., 2018; Polster et al., 2018), is required for establishing a functional link between Ca_V_1.1 and RYR1 (Perni et al., 2017). *Stac3*^−/−^ couplons have fewer Ca_V_ particles and fewer complete tetrads. To model the deletion parsimoniously, we ran the simulation with the parameter values listed in Table 1 as low, less affinity, modified by increasing every energy of feet with bound Ca_V_s (*a*_1_ to *a*_4_) by one unit, to decrease the affinity of the interaction. This simple change was adequate to simulate the decrease in average occupancy and *f*(4) observed in null muscles (Table 2).

## Online supplemental material

The supplemental material consists of four simulations that explore regions of parameter space other than those shown in the text to be consistent with the observed junctional images. The first three (Figs. S1, S2, and S3) illustrate parameter choices that remove one of the two key interactions. The simulation illustrated in Fig. S1 removes the steric effect, by making *S* = 0. The simulations in Figs. S2 and S3 preserve the steric effect and remove the allosteric effect. The simulation illustrated in Fig. S2 does it with a flat energy profile A, whereby *Δ E_o_* is the same, small and positive in all successive steps of increasing occupation. The one in Fig. S3 does it with a steep linear decay of energy; *ΔE_o_* is equal, large, and negative in successive steps. The simulation in Fig. S4 preserves the allosteric effect; the profile is monotonically decreasing, with progressively greater *ΔE_o_* (increasing affinity) as *o* increases. Note the difference with the modal profile of energies in the simulations presented in the main text. Other combinations of parameter values, which qualitatively span the parameter space, were also incompatible were the observations. Table S1 provides parameters of four versions of the model. Table S2 provides outcomes of simulations with parameters listed in Table S1.

## Discussion

The main tenet of our hypothesis is that the structure of triad junctions in skeletal muscle is maintained solely by interactions between the RYRs and Ca_V_s that form the couplon: the long-range order in the checkerboard emerges without recourse to ordered structures outside the couplon.

**Figure 7.**
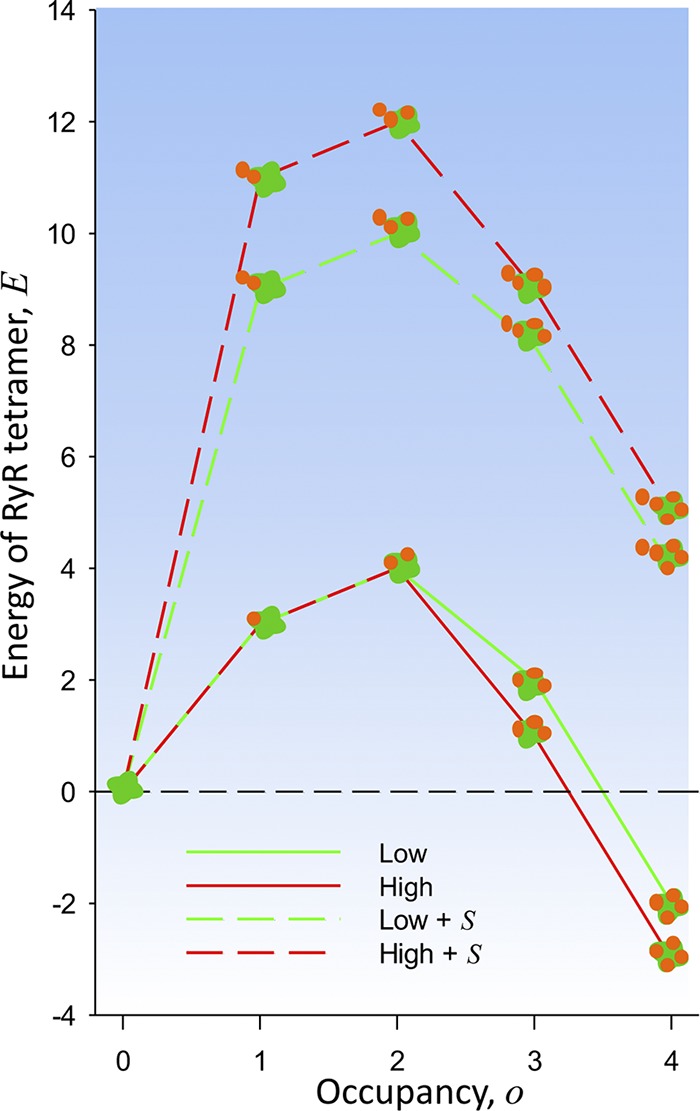
**Energy profiles consistent with observations.** Individual RYR tetramer energy (*E*) versus occupancy (*o*) for the low and high parameter sets as listed in Table 1. The large values at top of the graph (dashed lines) are energies in conditions of steric clash (occupancy of an adjacent protomer of an adjacent RYR tetramer). The observed configurations are those with occupancy 0, 3, and 4, found at *E* ≤ 2.

Although the hypothesis is formulated generally in terms of binding energies, the five adjustable parameters that implement it had to be constrained to a small region of parameter space in order to reproduce the arrangement of Ca_V_s in tetrads and the alternation of tetrads in the checkerboard. The suitable energy profiles are represented in Fig. 7 as four plots of couplon energies *E* versus *o*.

One of the energy features here found necessary had been proposed before: Protasi et al. (1997) noted that the outer borders of Ca_V_ complexes in tetrads exceed the contour of the underlying foot and invade the space where Ca_V_s would be located if attached to adjacent RYR channels. The span of tetrads and consequent overhang was specified further by Wolf et al. (2003). Consequently, Protasi et al. (1997) proposed that steric hindrance prevents occupancy by Ca_V_s of adjacent protomers in adjacent channels. As Protasi et al. (1997) also noted, steric hindrance alone does not explain full occupancy in alternating RYRs. Steric clashes can be avoided while overall occupancy is maintained, by having one to three Ca_V_s on every RYR tetramer (Protasi et al., 1997; example configurations with these properties documented in Fig. S1 and Table S1).

The other feature required to establish the observed structure is a dependence of the stability of the Ca_V_–RYR interaction on the occupancy of the other protomers in the same tetramer. Ca_V_ binding to a protomer was assumed to induce a conformational change that alters the binding properties of the other protomers in the same tetramer. The effect can propagate via the physical interface between the protomers. Considering that the individual Ca_V_s might interact with two underlying RYR protomers, the effect might also emerge from this putative three-body interaction.

The simplicity of the model afforded an extensive exploration of the parameter space. The three choices of parameters documented in Tables 1 and 2 demonstrate the properties in the presence of steric and allosteric effects. Positive cooperativity is realized by *a*_o_ energies that decrease with occupancy. Same as in classic Monod-Wyman-Changeux allosteric models (Monod et al., 1965), the last binding step, leading to the full occupation of a foot by a Ca_V_ tetrad, had to be especially rewarded. Schemes with more gradual and evenly spread cooperative features, as the example in Fig. S4, did not match the observations.

As represented in Fig. 7, the allowed energies *a*(*o*) of an RYR tetramer have two wells, at *o* = 0 and 4, separated by a barrier at intermediate occupancies. Another binary interaction, steric clash, is then sufficient to establish the longer-range checkerboard periodicity. Furthermore, to fit the data, the dependence *a*(*o*) cannot be symmetric; the well at high occupancy must be deeper than the one at low occupancy. This asymmetry is required to offset the repulsive effect of the impending steric clash, the likelihood of which increases at high values of *o*.

The examples named low and high differ in the steepness of the decrease of energy at high occupancy. In both cases the outcomes match the observations qualitatively. The good quantitative fit of the high model suggests that the allosteric effect amounts to energy changes of ∼2 *kT* per site as occupancy increases. The model requires at least 6 *kT* of energy in the steric clash. The value is reasonable in view of the profiles in Fig. 7; indeed, this penalty makes the probability of the fully occupied foot with occupied adjacent foot—a configuration not seen in actual junctions—about equal to that of a foot with occupancy 1 or 2, also rare in actual structures. The frequently visited configurations span a range of no more than 4 *kT* per RYR tetramer.

The main conclusion (positive cooperativity favors full occupancy of RYR tetramers, and steric clash prevents it in neighbors) may seem tautological. The revelation, though, is that these simple assumptions at the tetramer level are sufficient to impose long-range regularity and asymmetry in couplons of any size.

The present simulations provide an additional insight. Ever since Block et al. (1988) demonstrated the checkerboard structure, the functional role of C channels has been puzzling. The initial proposal that C channels are activated by calcium released via V channels (i.e., CICR; Ríos and Pizarro, 1988) was later dismissed (Figueroa et al., 2012; reviewed by Ríos, 2018). The question is now whether C channels activate at all or might just work as spacers, needed to maintain the couplon structure. Showing that a conversion of C to V channels is possible, the present simulations suggest that C RYRs constitute a structural reserve, channel proteins that can intermittently adopt a functional role, perhaps prolonging the natural cycle of the array.

### Limitations and further hypothesis testing

The most severe limitation of the present comparison lies with the analysis of images. As described, we start from the assumption that a couplon is being imaged. This implies assuming the presence of an ordered array of RYRs, which is never seen; its presence is only inferred from that of Ca_V_s forming tetrads, complete or incomplete. Even when applied correctly, this method is skewed toward the best assembled and most complete sets of tetrads, which favors an agreement with the canonical structure. Correcting this problem will require simultaneous imaging of RYRs and Ca_V_s, which is not possible with conventional electron microscopy. A way forward is suggested by multicolor superresolution microscopy, which allowed defining the spatial relationship of RYR with other couplon molecules in cardiac myocytes (Jayasinghe et al., 2012, 2018) and skeletal myofibers (Jayasinghe et al., 2014) at a resolution suitable to define individual RYR tetramers. However, simultaneous imaging of RYR and Ca_V_ arrays has not yet been achieved for skeletal muscle.

The hypothesis is thermodynamic, concerned with structure stability rather than the dynamics of assembly. Indeed, the outcomes of Metropolis–Hastings simulations are determined by the energies of the available configurations; these are not simulations in time. While it would be easy to include kinetics in the model, a comparable experimental database would require a combination of static imaging with measures of molecular turnover times or dynamic imaging of junctional structures. As an example, the gradual formation of couplons in differentiating cultured BC_3_H1 cells (Protasi et al., 1997) would provide opportunities for dynamic imaging of live preparations in the process of association between developing arrays of RYRs and Ca_V_1.1.

The hypothesis is not molecular. By RYRs or Ca_V_s we refer to the particles visible with electron microscopy. These particles are multimeric and incorporate smaller proteins. The fine molecular grain, whereby the presence of some components may change the interaction properties, is not addressed here. As a related limitation, the hypothesis does not specify concentrations or chemical potentials of the interacting particles.

There are paths to a molecular formulation; for an example we took advantage of the approach of Linsley et al. (2017), whereby stac3 is suppressed. Stac3 binds to Ca_V_1.1 and is required for the functional interaction between RYR1 and Ca_V_1.1 (Perni et al., 2017; Polster et al., 2018). Its presence enhances the heterologous expression and assembly into working couplons of essential EC coupling proteins in *Xenopus* oocytes (Wu et al., 2018). Linsley et al. (2017) found reduced occupancies by Ca_V_1.1 particles in junctions of *stac3*^−/−^ muscles. The present simulations reproduced the observations via a simple reduction in affinity. Other interpretations of the effect of stac3 can be envisioned. For example, the presence of the protein could increase the incorporation of Ca_V_ to junctional membranes. Measures of this incorporation would justify including particle concentration and chemical potential as explicit variables in the model. Other animal models, with mutations or deletions of molecular domains putatively critical for the interaction, could be imaged for further casting of the hypothesis in molecular terms.

### Alternative models

Given the present limitations, the agreement of these simulations with the available data does not preclude other interaction schemes. Two alternatives deserve consideration. One keeps the premise that interactions are limited to particles within the couplon but extends their range, allowing the binding energies to be modified by occupancy of every protomer in adjacent tetramers, not just the adjacent one. The physical connection of the array provides the substrate necessary for such extended allosteric interactions. A second possibility is to break the couplon limitation—the basic tenet of the present hypothesis—assuming instead that the alternation of occupied and free feet is imposed by a larger structure. The membrane-associated periodic skeleton, recently demonstrated in neurons by super-resolution imaging, is an example of spatial regularity of a frequency similar to that of the junctional checkerboard, imposed in this case by the cytoskeleton (Xu et al., 2013; D’Este et al., 2016). While these and other alternatives are plausible, our working model remains the simplest one consistent with the observations.

## Conclusions

The main outcome of this study is an answer to the question of whether a couplon with a stable checkerboard of Ca_V_1.1 can be sustained by binary binding between the two major couplon members. The answer is yes, provided there are strong allosteric-cooperative effects within an RYR tetramer that favor full occupancy by Ca_V_1.1 and an even stronger steric clash when Ca_V_s bind to adjacent protomers in adjacent tetramers. The study does not clarify the molecular aspects of these interactions and does not and cannot exclude more complex interactions within the couplon or the participation of proteins of the cytoskeleton. The quantitative comparison of model outcomes with actual observations is hampered but not totally interdicted by technical limitations in the latter, chiefly the current impossibility to image the T tubular/plasmalemma and the SR sides of the same junction and some problems with the freeze-fracture technique. Location microscopy could open a path to imaging Ca_V_s and RYRs associated in a junction. Dynamic imaging of live preparations would be necessary to provide observations on binding kinetics. As a bonus, the bistability exhibited by the successful outcomes suggests a function for C channels, revealing the possibility that they become active in calcium release, by role reversal with their V neighbors.

## Supplementary Material

Supplemental Materials (PDF)

## Footnotes

1 Combining the evidence from cryo-EM and freeze-fracture images, it is suggested that Ca_V_1.1 particles interact with RYR at domains slightly offset from the corners in the roughly square profile of the RYR tetramer (e.g., Zalk et al., 2015; Samsó, 2017). While some of the interacting stretches have been identified (Nakai et al., 1998), there is no evidence that the interaction is limited to one RYR protomer. Identifying the Ca_V_1.1 binding partners in RYR1 as protomers and schematically placing the bound Ca_V_s at corners of the RYR tetramer are simplifications. “Binding sites” or “binding partners” may be substituted for “protomers,” with no consequences for the present simulations.

2 The simulation procedure forbids direct vertical transitions. The connection between states of the same occupancy must instead proceed in two stages. For example, the state of occupancy 2 with two simultaneously occupied adjacent protomers may not directly transition to occupancy 2 with one adjacent occupied protomer; it must first transition to occupancy 1 (with one adjacent occupied protomer).

3 This number, close to 2^24^, is much smaller than the number of possible configurations, $2^{n_{p}}$, where *n_p_* is the number of RYR protomers in the couplon, 96 in the present case. This is consistent with the fact that in large ensembles, sample averages converge rapidly to their population values. Additionally, with the values of *S* favored in the present tests, the configurations with simultaneous occupancy of adjacent protomers were essentially forbidden, which greatly reduced the accessible state space.
