# Supplementary material for: The binding interactions that maintain excitation–contraction coupling junctions in skeletal muscle
Source: J Gen Physiol. 2019 Feb 6;151(4):593–605. doi: 10.1085/jgp.201812268 (PMC6445584; doi:10.1085/jgp.201812268)
Supplement: Supplemental Materials (PDF) [file JGP_201812268_sm.pdf]

Supplemental material

Ríos et al., https://doi.org/10.1085/jgp.201812268

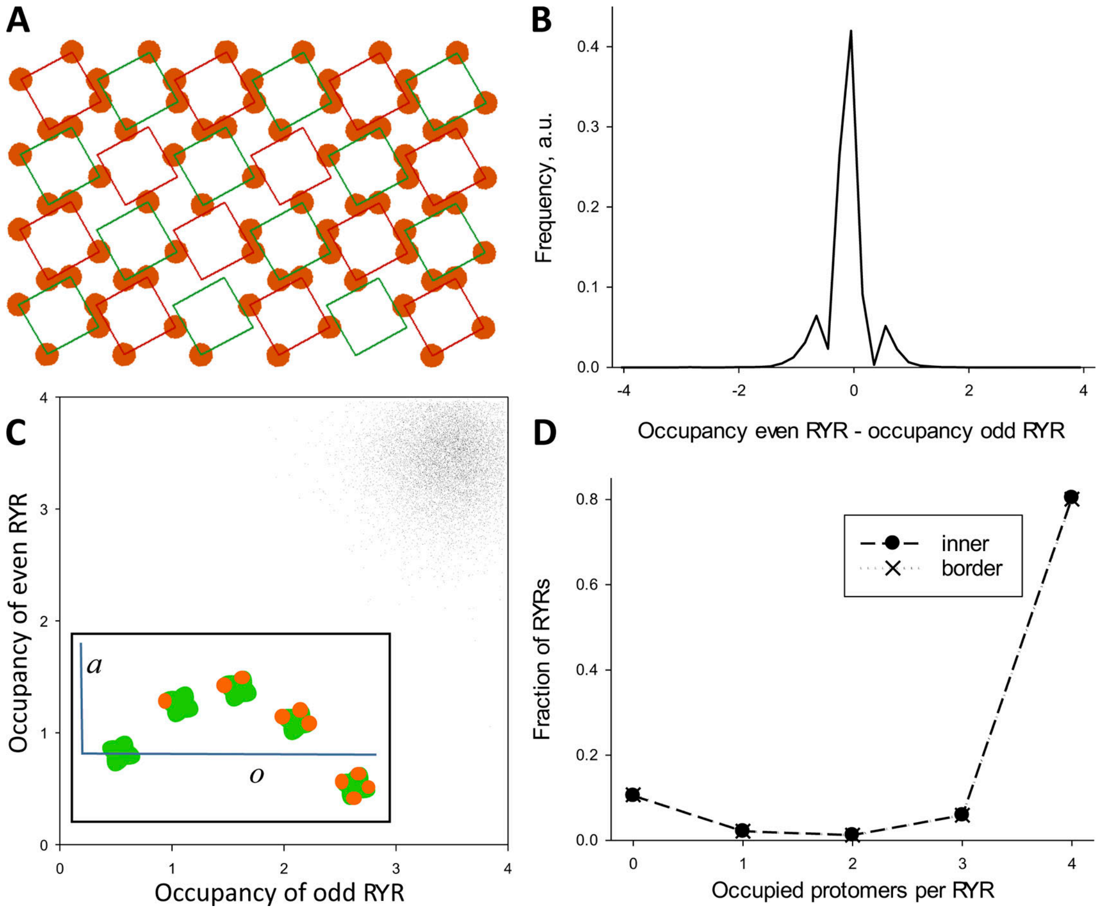

Figure S1. **Simulation outcomes for a model that removes the assumption of steric hindrance (parameter *S* = 0).** Parameter set A_a in Table S1. **(A)** Configuration of a couplon of four rows and six columns, representative of configurations favored under this condition. Same as in Fig. 2; green and red squares represent even and odd channels, respectively (RYR tetramers). Note many adjacent protomers simultaneously occupied. **(B)** Distributions of bias and difference between couplon occupancy of even and odd channels (definitions in Eq. 8). The average asymmetry calculated from this distribution is 0 (Eq. 9; numerical outcomes listed in Table S1). The absence of a steric effect eliminates bias and asymmetry. **(C)** Couplon occupancies (co_e versus co_o) in a sample of 2×10^7 configurations. **(D)** Fractional occupancies *f*(*o*) for both sets of parameters. Crosses plot fractional occupancies of border feet (i.e., rows 0 and 3 and columns 0 and 5); circles represent fractional occupancies of inner feet. The absence of a steric effect trivially abolishes the differences between inner and border feet.

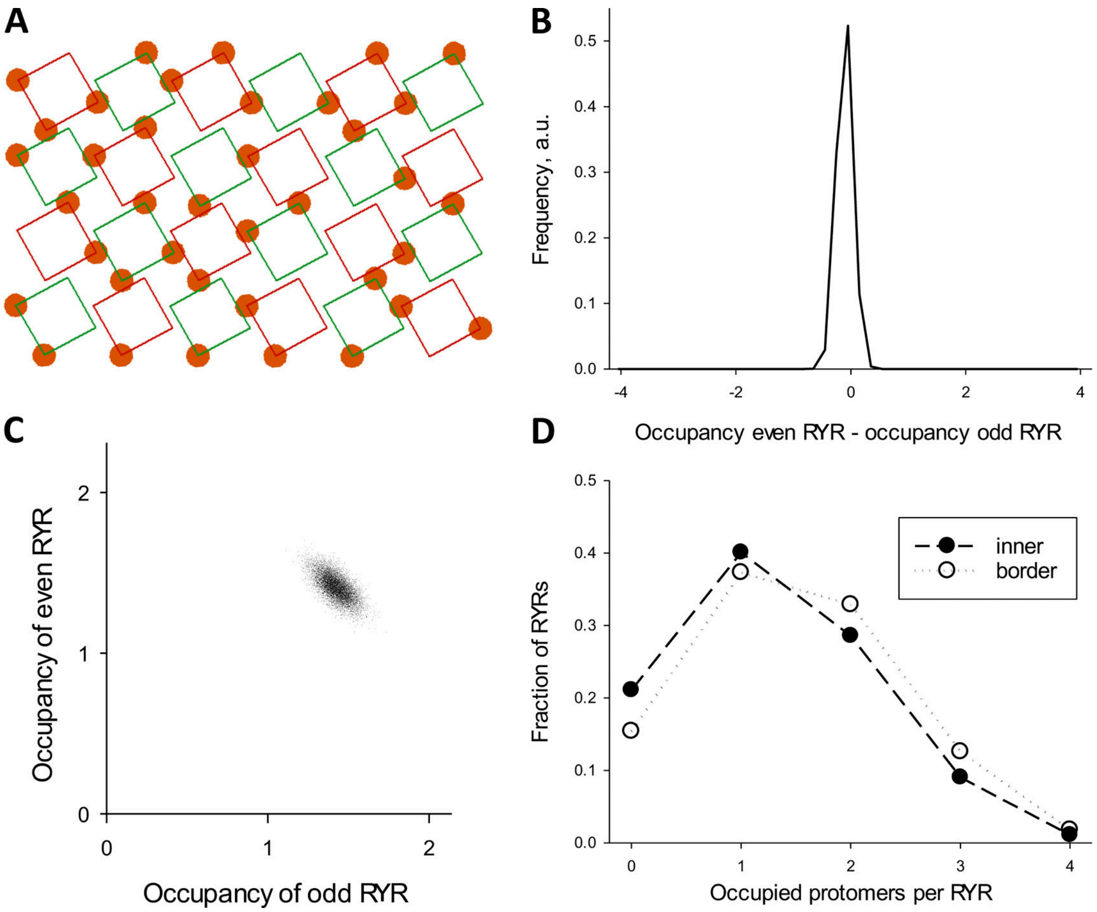

Figure S2. **Outcomes for a model that removes the allosteric effects (the elements in vector A—the energies of a tetramer at increasing levels of occupancy—are spaced by an equal difference *ΔE*, the binding energy for all protomers, regardless of occupancy).** Energies listed as vector A_b in Table S1. The *ΔE* are small and positive, corresponding to a midrange affinity. **(A)** Representative configuration of a 4 × 6 couplon. Green and red squares represent even and odd channels, respectively. **(B)** Distribution of bias. The average asymmetry calculated from this distribution is close to 0 (Table S1). **(C)** Occupancies (co_e versus co_o) in a sample of 2 × 10^7 configurations. Occupancies stay similar for even and odd channels at values that depend on the chosen *ΔE*. **(D)** Fractional occupancies *f*(*o*) for both sets of parameters. Open circles plot fractional occupancies of border feet; filled circles represent fractional occupancies of inner feet. In the present case, with *ΔE* small and positive, the most favored values of occupancy *co* remains below 2. The outcomes of all simulations are listed in Table S1.

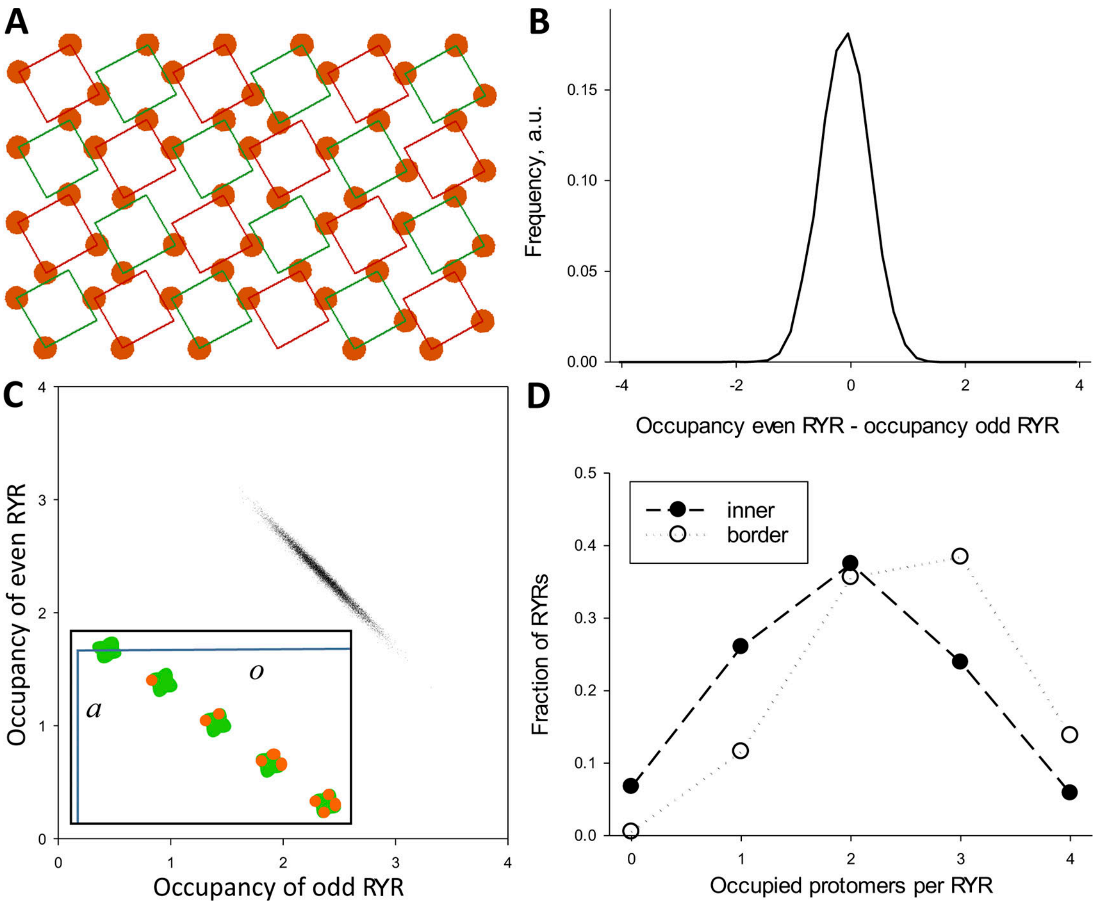

Figure S3. **Outcomes for a model that removes allosteric effects, with equal high binding affinity at all levels of occupancy.** The energies of a tetramer at increasing levels of occupancy (vector A_c in Table S1) are spaced by an equal large negative difference ΔE, which determines a large binding affinity for all protomers, regardless of occupancy. **(A)** Representative configuration of a 4 × 6 couplon. **(B)** Distribution of bias. The average asymmetry calculated from this distribution is close to 0 (Table S1). **(C)** co_e versus co_o in a sample of 2×10^7 configurations. Occupancies are similar for even and odd channels. **(D)** Fractional occupancies $f(o)$ for both sets of parameters. Open and filled circles plot fractional occupancies of border and inner RYR tetramers, respectively. In the present case, with ΔE large and negative, the most favored values of occupancy *co* increase with regard to the previous example (Fig. S2), and the increase is especially marked in border RYR tetramers, where the steric repulsion is lower.

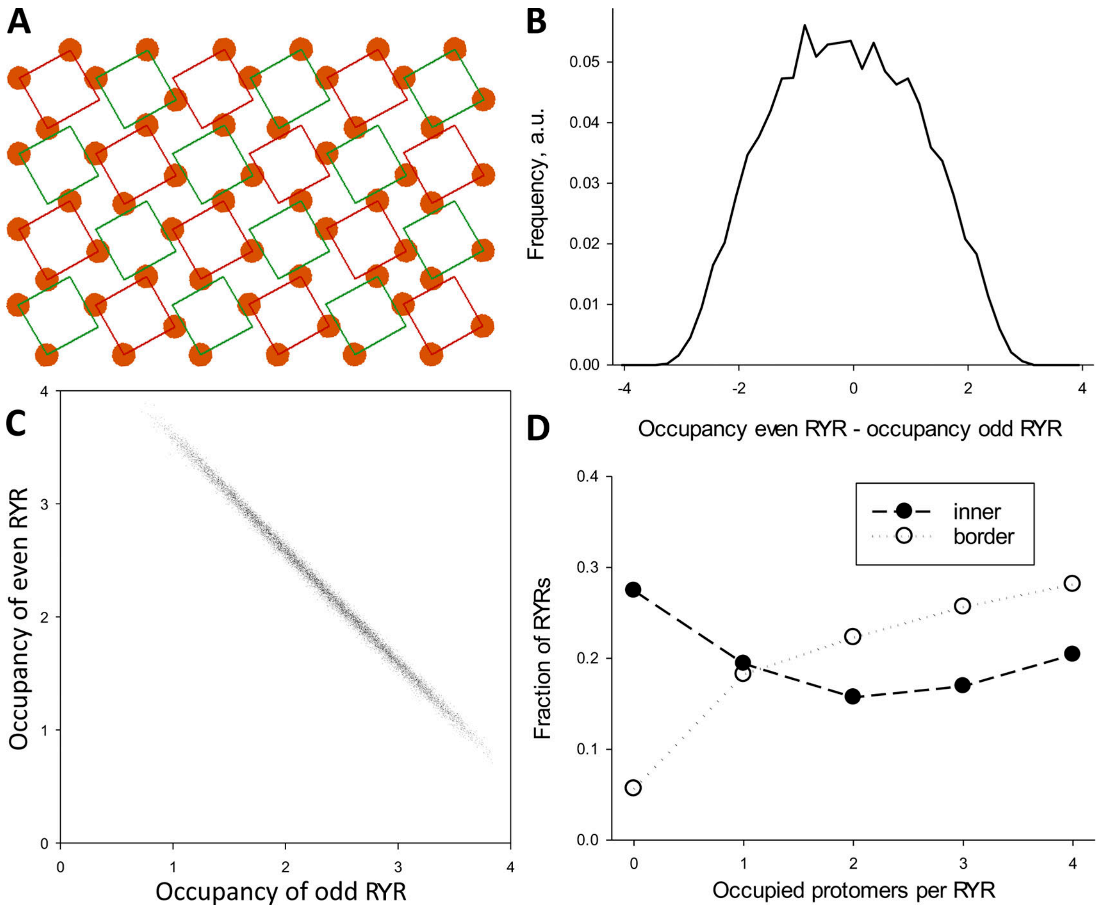

Figure S4. **Outcomes for a model that includes allosteric effects, with equal increases of binding affinity at successive levels of occupancy.** The energies of a tetramer at increasing levels of occupancy (vector A_d in Table S1) are spaced by an increasing negative ΔE, which determines equal increases in binding affinity as successive protomers become occupied, an equal allosteric effect at every level of occupancy. **(A)** Representative configuration of a 4 × 6 couplon. **(B)** Distribution of bias, which is much wider than in the other cases illustrated in Online supplemental material, reaching an average asymmetry of 1.15 (Table S1). **(C)** co_e versus co_o in 2 × 10^7 configurations. Occupancies of even and odd channels vary widely but lack the preference for high asymmetry (or bistability) exhibited by the simulations in Fig. 2. **(D)** Fractional occupancies *f*(*o*). In the present case, the higher levels of occupancy are more frequent, although the tendency is opposed by the steric effect, which hinders occupancy at inner feet. This effect is especially marked in this four-row couplon, in which high occupancy (*o* = 3 or 4) of border feet will severely hinder occupancy of the two rows of inner feet. Given this choice of allosteric parameters, the steric effect imposes an asymmetry between inner and outer feet, rather than the canonical asymmetry between even and odd feet.

Table S1. **Parameters of four versions of the model**

	**Occupancy**					***S***
	**0**	**1**	**2**	**3**	**4**	
**Energy per RYR tetramer**						
A_a	0	3	4	2	−2	0
A_b	0	0.1	0.2	0.3	0.4	8
A_c	0	−3	−6	−9	−12	8
A_d	0	−1	−3	−6	−10	8

The different rows lists vectors (A) of the values a_0 of energies of the RYR tetramer at its five values of occupancy. The last column list the value *S* of the energy penalty incurred by steric clash. A_a is the set listed as high in Table 1 combined with the assumption of no steric hindrance (illustrated in Fig. S1). A_b is a case with no allosteric interactions and low site affinity (Fig. S2). A_c is a case with no allosteric interactions and high site affinity (Fig. S3). For A_d, allosteric effect is present at all levels of occupancy (the negative ΔE, which sets binding affinity, increases by the same amount, 1 unit, at each successive level of occupancy; Fig. S4).

Table S2. **Outcomes of simulations with parameters listed in Table S1**

	Allosteric energies (A)			
	A_a	A_b	A_c	A_d
Average occupancy	3.43	1.15	2.34	2.29
Average occupancy inner feet	3.44	1.29	1.96	1.83
Average occupancy border feet	3.42	1.48	2.53	2.52
Full occupancy	0.8	0.02	0.12	0.25
Average asymmetry	0.46	0.14	0.44	1.12

Average occupancy is *co* (Eq. 7) averaged over the configurations reached in 2×10^7 transitions. Full occupancy is the variable *f*(4). Average asymmetry is the average over the same 2×10^7 configurations of the variable *y* (Eq. 9). Cases A_a to A_c illustrate that removing either the steric or the allosteric interactions crucially reduces asymmetry of occupancy of (even and odd) RYR channels. The outcomes with A_d, which implements a strong allosteric effect at all occupancy levels, include a greater asymmetry but no particular preference for the extremes of occupancy (0 and 4) generated with the preferred sets. Illustrations and additional details are given in Figs. S1, S2, S3, and S4.
